# Development of salutogenetic factors in mental health - Antonovsky’s sense of coherence and Bandura’s self-efficacy related to Derogatis’ symptom check list (SCL-90-R)

**DOI:** 10.1186/1477-7525-11-80

**Published:** 2013-05-08

**Authors:** Henrik Kröninger-Jungaberle, Dennis Grevenstein

**Affiliations:** 1 Institute of Medical Psychology, Centre for Psychosocial Medicine, University Hospital Heidelberg, Bergheimer Str. 20 69115, Heidelberg, Germany; 2 Psychological Institute, University of Heidelberg, Hauptstraße 47, 69117, Heidelberg, Germany

**Keywords:** Resilience - mental health, Salutogenesis, Sense of coherence, Self-efficacy, Symptom-check list (SCL-90), Longitudinal development, Cross-lagged design

## Abstract

**Background:**

The paper analyses how resilience factors and mental health problems interrelate in a 3-year-longitudinal study with 16–19 year olds.

**Methods:**

Resilience was measured with a 13-item short version of the Life-Orientation-Scale by Antonovsky (sense-of-coherence, SOC) and a 10-item self-efficacy-scale (SWE) by Jerusalem and Schwarzer. Mental health problems were measured with Derogatis Symptom Check list (SCL-90-R). The data set included 155 participants and was analyzed using Structural Equation Modeling (SEM) designed to examine mutual influence in longitudinal data with Mplus software.

**Results:**

The descriptive data analysis indicates (1) negative correlations between SOC and SCL-90-R at both age 16 and 19 in all subscales but somatization and likewise (2) between self-efficacy and SCL-90-R. (3) SOC correlates positively with SWE at age 16 and 19.

Results of SEM analysis were based on the assumption of two latent variables at two points in time: resilience as measured with mean SOC and mean self-efficacy scores and health problems measured with sub scale scores of SCL-90-R – both at ages 16 and 19. The first SEM model included all possible paths between the two latent variables across time. We found (4) that resilience influences mental health problems cross-sectionally at age 16 and at age 19 but not across time. (5) Both resilience and mental health problems influenced their own development over time. A respecified SEM model included only significant paths. (6) Resilience at age 16 significantly influences health problems at age 16 as well as resilience at age 19. Health problems at age 16 influence those at age 19 and resilience at age 19 influences health problems at age 19.

**Conclusion:**

(a) SOC and self-efficacy instruments measure similar phenomena. (b) Since an influence of resilience on mental health problems and vice versa over time could not be shown there must be additional factors important to development. (c) SOC and self-efficacy are both very stable at 16 and 19 years. This refutes Antonovsky’s assumption that SOC achieves stability first around the age of 30. SOC and self-efficacy are protective factors but they seem to form in (early) childhood.

## Background

The salutogenic approach to health aims to explain how people remain healthy, rather than how people get sick. Salutogenesis is part of a family of theories that are also discussed as resilience. Fundamental in salutogenesis is to consider health as a position on a health ease/dis-ease continuum. Being healthy is not considered a merely physiologically rated on/off-state, but a result of an ongoing movement towards the health end of that continuum. At the core of this salutogenic theory lies the sense of coherence (SOC) construct. Antonovsky [1-3] described sense of coherence as a global health-protective life orientation. The construct includes three interrelated components: *comprehensibility* describes the belief that the world is comprehensible, consistent, predictable and explicable. *Manageability* means a belief that a person is able to deal with the demands of life and has sufficient resources to meet internal and external stimuli. Finally, *meaningfulness* describes the belief that challenges are worthy of investment and engagement. Those three factors are supposed to represent a basis for successful coping with stressors and thus for remaining healthy.

The positive influence of SOC has been examined in the past. Various studies have shown associations between SOC and various health related measures [4]. In adults SOC has been linked to general psychological well-being [5], or negatively linked to burnout [6], depression and anxiety [7-9] as well as multiple other health behaviors [10]. This also holds true for clinical populations [11-13].

It has also been shown that SOC is a valuable construct for children and adolescents. SOC was found to indicate lower levels of anxiety, depression and ways of coping with anxiety [14,15].

Collecting more evidence about the development of the SOC is important for constructing future interventions. According to Antonovsky [1], the foundation for a high SOC in adulthood is laid in childhood and early adulthood by positive life experiences. It is therefore possible that past psychological problems influence the current level of SOC in later adulthood. Building on Antonovsky’s early theories, SOC is expected to remain relatively stable throughout adult life, yet there are at least two different hypotheses about the life development or changes of SOC [1]. The *age-hypothesis* expects that SOC may be fluctuant at a younger age, but is stable in later years. A person who has once developed a certain level of SOC after the age of around 30 is expected to remain at this level or, if changes in the light of life crises occur, return to his or her level of SOC soon. In contrast, changes in SOC would last much longer or even be permanent if they occur in childhood or early adulthood. The *level-hypothesis* expects that individuals who arrive at a high level SOC in adulthood are less likely to show a change in SOC later. Individuals with a lower level of SOC may show less stability. If for example a person with a high level of SOC experiences challenging situations, she/he is expected to be better prepared to deal with problems and retain her positive attitude towards life and its challenges. On the other hand, a person with a lower level of SOC may not be able to cope with difficult situations and thus lose faith in the world and herself even more which would indicate an even further drop in SOC. Thus, SOC should generally be more fluctuant at a younger age.

In many formalized models of health psychology, self-efficacy or one’s own belief to control and succeed in a given situation plays an important role [16-18]. Self-efficacy [17,19] has been defined as a belief in one’s own abilities to succeed in specific situations. A person’s sense of self-efficacy can play a major role in the assessment of goals, tasks, and challenges and how to approach them. With a high degree of self-efficacy one is expected to view difficult or new tasks as challenges to be mastered rather than problems to be avoided. The construct has been shown as a moderator or mediator in many different areas of health psychology from addictive behaviors [20-22], physical activity and depression [23] to coping with cancer [24]. Self-efficacy has therefore also been considered a resilience factor [25].

Still, there is ongoing discussion about whether sense of coherence and self-efficacy are really protective factors or whether they are merely a result of being healthy. Most studies that have shown a cross-sectional correlation between health and various resilience factors fail to answer that classical hen-and-egg question. In the present research a longitudinal cross-lagged design is used to examine the mutual influence of those constructs.

## Methods

### Study sample

The following study was part of a 10-year-longitudinal study of resilience and drug usage (RISA) conducted in the area of Heidelberg from 2003 to 2012. The study was approved by the ethics committee of the University Hospital Heidelberg (No. 218/2005). Participants were 318 students of four different schools with a mean age of 14 at the beginning and a mean age of 24 at the end of the study. All in all the study comprised 14 data collection events. While SOC-13 was provided 14 times in the course of ten years, SLC-90-R was only provided twice (at the age of 16 and 19) within the 10-years-data collection comprising a larger set of research instruments. Thus merely data at ages 16 and 19 could be used for the present study. A subsample of 155 participants (61 male and 94 female) had completed the required questionnaires at ages 16 and 19. 46.5% of the participants (N = 72) grew up in a traditional family, which was considered as living with both biological parents up to the age of 18 years. The German school system provides three different secondary school options after completing primary school. Ranking the school types from lowest to highest, students commonly finish the “Hauptschule” at the age of 15 to 16, “Realschule” at age 16 to 17 and “Gymnasium” at age 18 to 19. Participants were distributed almost equally across the three different school types. Table 1 provides an overview of sample characteristics.

**Table 1:** Sample characteristics

Sex	61 male (39.4%)	94 female (60.6%)	
Type of school	Hauptschule: 45 (29%)	Realschule: 38 (37.4%)	Gymnasium: 52 (33.5%)
Traditional family	72 yes (46.5%)	83 no (53.5%)	

### Measures

#### SOC measured with life orientation scale

A German 13 item short adaption of Antonovsky’s original Orientation to Life Questionnaire [26] was used to measure sense of coherence. A later version was evaluated by Schumacher et al. [27]. It is accepted as a valid and reliable (Cronbach Alpha = .85) measure of the construct. It includes four meaningfulness items (e.g. “Do you have the feeling that you don’t really care about what goes on around you?”), five comprehensibility items (e.g. “Has it happened in the past that you were surprised by the behavior of people whom you thought you knew well?”) and four manageability items (e.g. “Has it happened that people whom you counted on disappointed you?”). In contrast to the original scale, answers were given on a 5 point scale rather than a 7 point scale which is the result of using an earlier adaptation throughout a 10-year longitudinal study.

In our sample, Cronbach’s Alpha was .886 at age 16 and .883 at age 19. Even though Antonovsky theoretically claimed a three factor structure for the sense of coherence scale, we were not able to reproduce three factors. Similar findings have been reported before, especially for short adaptations [7,27-29]. In the final analysis, we used a mean score for the whole SOC construct, instead of the individual dimensions. For comparison only, we also computed sum scores and adjusted those to the original 7 point scale by multiplying with 1.4. Please note however, that no statistical analysis was done with those sum scores. At age 16, participants had a mean adjusted sum score of 65.22 (SD = 11.55) with only a very small increase to 66.48 (SD = 10.81) at age 19. Table 2 also lists scores separated for men and women. These scores are very similar to those published by Schumacher et al. [27] who found a mean SOC of 67.31 (SD = 12.09) for men ages 18 to 40 and 64.52 (SD = 11.61) for women ages 18 to 40.

**Table 2:** Means and standard deviations of the study variables for women (n = 94) and men (n = 61)

**Variable**	**Women M (SD)**	**Men M (SD)**	**T value**	**P value**
SOC 16 (mean) (sence of coherence)	3.51 (0.62)	3.69 (0.64)	1.71	.088 (n.s.)
SOC 16 (adjusted sum score)	63.99 (11.35)	67.18 (11.68)		
SOC 19 (mean) (sence of coherence)	3.61 (0.60)	3.71 (0.58)	1.09	.277 (n.s.)
SOC 19 (adjusted sum score)	65.74 (10.91)	67.65 (10.63)		
SWE 16 (self efficacy)	2.86 (0.34)	2.96 (0.38)	1.71	.088 (n.s.)
SWE 19 (self efficacy)	2.97 (0.37)	3.00 (0.35)	0.51	.612 (n.s.)
SCL SOM 16 (Somatization)	7.43 (7.02)	6.77 (5.64)	- 0.62	.534 (n.s.)
SCL O-C 16 (Obsessive-Compulsive)	5.90 (5.33)	6.09 (5.60)	0.22	.827 (n.s.)
SCL I-S 16 (Interpersonal Sensitivity)	5.38 (5.13)	4.52 (4.71)	- 1.06	.291 (n.s.)
SCL DEP 16 (Depression)	7.33 (7.35)	6.10 (6.48)	- 1.08	.282 (n.s.)
SCL ANX 16 (Anxiety)	4.68 (5.32)	3.89 (4.36)	- 0.99	.324 (n.s.)
SCL HOS 16 (Hostility)	3.82 (3.64)	3.64 (3.52)	- 0.28	.778 (n.s.)
SCL PHOB 16 (Phobic Anxiety)	1.10 (2.17)	0.99 (1.70)	- 0.33	.741 (n.s.)
SCL PAR 16 (Paranoid Ideation)	3.40 (3.62)	3.51 (3.73)	0.17	.861 (n.s.)
SCL PSY 16 (Psychoticism)	2.90 (4.57)	3.76 (5.34)	1.05	.297 (n.s.)
SCL SOM 19 (Somatization)	6.73 (6.11)	5.39 (4.09)	- 1.667	.097 (n.s.)
SCL O-C 19 (Obsessive-Compulsive)	5.47 (5.55)	6.43 (5.50)	1.06	.288 (n.s.)
SCL I-S 19 (Interpersonal Sensitivity)	5.18 (5.55)	4.99 (4.95)	- 0.21	.831 (n.s.)
SCL DEP 19 (Depression)	7.06 (8.08)	6.04 (6.88)	- 0.84	.405 (n.s.)
SCL ANX 19 (Anxiety)	3.94 (5.33)	4.02 (3.37)	0.12	.908 (n.s.)
SCL HOS 19 (Hostility)	3.20 (3.49)	3.34 (2.78)	0.26	.794 (n.s.)
SCL PHOB 19 (Phobic Anxiety)	1.21 (2.49)	0.73 (1.74)	- 1.33	.186 (n.s.)
SCL PAR 19 (Paranoid Ideation)	3.32 (4.18)	3.55 (3.26)	0.36	.719 (n.s.)
SCL PSY 19 (Psychoticism)	2.96 (5.06)	2.88 (3.70)	- 0.11	.915 (n.s.)

#### Self-efficacy measured with SWE scale

The SWE self-efficacy scale was developed by Jerusalem und Schwarzer [30,31] and consists of 10 items. Questions include “If there are challenges, I can find a way to succeed” and “I can find a solution for every problem” (translation by the authors). Answers were given on a 4 point scale labeled “not true”, “rarely true”, “mostly true” and “completely true”. The reliability of the scale is considered sufficient (Cronbach’s Alpha = .79).

In our sample, Cronbach’s Alpha was .850 at age 16 and .849 at age 19. For the final analysis we computed mean scores. In all the completed SWE questionnaires, there was a total of 0.57% of values missing.

#### Psychological symptoms measured with SCL-90-R

The Symptom Checklist-90-R (SCL-90-R) [32] is a widely used instrument to measure a broad range of psychological problems and symptoms of psychopathology. A revised German version was produced by Franke [33,34]. It consists of 90 items and the following 9 different subscales: SOM – Somatization, O-C - Obsessive-Compulsive, I-S - Interpersonal Sensitivity, DEP – Depression, ANX – Anxiety, HOS – Hostility, PHOB - Phobic Anxiety, PAR - Paranoid Ideation, PSY – Psychoticism. It provides three global scores as well as sum scores for the subscales. The reliabilities (Cronbach’s Alpha) of the different subscales range from .75 to .97.In our sample reliabilities ranged from (Alpha) .748 to .868 at age 16 and from .722 to .889 at age 19.

In the present research we were mostly interested in the mutual influence of the constructs, rather than individual diagnostics. Even though they are intercorrelated, the different subscales of the SCL-90-R provide a possible measure for many different aspects of psychopathology. Therefore we chose to use the subscales directly instead of the global scores, for both correlations and SEM analysis.

The standard procedure is to compute sum scores for each subscale. As those scores were severely skewed we added a scalar of 1 to avoid plain 0 scores and then used a logarithmic transformation to get normally distributed scores for the SEM analysis.

#### Statistical analysis

The descriptive data analysis was carried out using SPSS 19. The Structural Equation Modeling (SEM) analysis was carried out using Mplus 5.21 [35]. We examined the relationship between the described resilience factors and mental health by using SEM. With this method we can model the mutual influence in longitudinal data. In SEM all the study variables can be investigated at the same time in the same model, so it is possible to model interconnections and mutual influence of the variables as well as modeling the development of variables over time. SEM also allows the modeling of latent variables which can represent larger constructs. Those latent variables are not measured directly, but are estimated from directly measured manifest variables. SEM is primarily based on model fitting and estimation. The whole model can then be estimated whether it accurately represents the empirical data. The goodness-of-fit of the models was evaluated using the (1) χ^2^ test (a good fit is indicated by non-significant values), (2) the comparative fit index CFI (values of 0.90 and above indicate a good model fit) and (3) the root mean square error of approximation RMSEA (values of 0.05 and less indicate a good model fit, values 0.06 to 0.08 indicate a reasonable fit and values close to 0.10 a poor model fit). A Maximum Likelihood estimator was used for parameter estimation.

## Results

### Descriptive data analysis

We started with a cross-sectional data analysis. First we checked for gender differences in the SOC, SCL and SWE scores. None of the variables differed significantly in our sample (see Table 2 for means).

Most of the variables were highly intercorrelated. For women, SOC showed correlations with the SCL-90-R subscales between - .461 and -.692 at age 16 and between - .380 and - .653 at age 19. For Men, correlations of SOC and SCL-90-R subscales are between – .401 and - .678 at age 16 and between – 1.79 and - .676 at age 19. With the notable exception of somatization at age 19 all the correlations are significant.

SWE significantly correlates with the SCL-90-R subscales of O-C (− .295), I-S (−.300), DEP (−.309), ANX (−.286), HOS (−.242), PAR (−.214) and PSY (−.244) for women at age 16. At age 19 there are correlations with the subscales of I-S (−.264), DEP (−.199), ANX (−.246), PAR (−.222) and PSY (−.241). For Men, SWE correlates significantly with all the SCL-90-R subscales except phobic anxiety at age 16 between -.280 and -.470. At age 19, SWE correlates with O-C (−.257), I-C (−.345), DEP (−.364) and ANX (−.364).

SOC correlates with SWE .555 at age 16 and .509 at age 19.

Across time, SOC at age 16 correlates with SOC at age 19 .635. SWE at age 16 correlates with SWE at age 19 .616. The SCL-90-R subscales correlate from age 16 to age 19 between .324 and .706.

This provides evidence that SOC and SWE are indeed highly related constructs. Both constructs show negative correlations with most subscales of the SCL-90-R.

### Structural equitation modeling (SEM)

We assumed two latent variables at two different points in time, the construct of resilience as measured by the mean SOC and mean SWE scores and the construct of mental health as measured by the log transformed sub scale scores of the SCL-90-R. Both constructs were modeled for the ages 16 and 19. Because of theoretical implications, we generally assumed a directed influence of resilience factors on health rather than assuming a simple covariation. As we couldn’t find any gender differences in the descriptive data analysis we did not include gender in the SEM.

**Figure 1:**
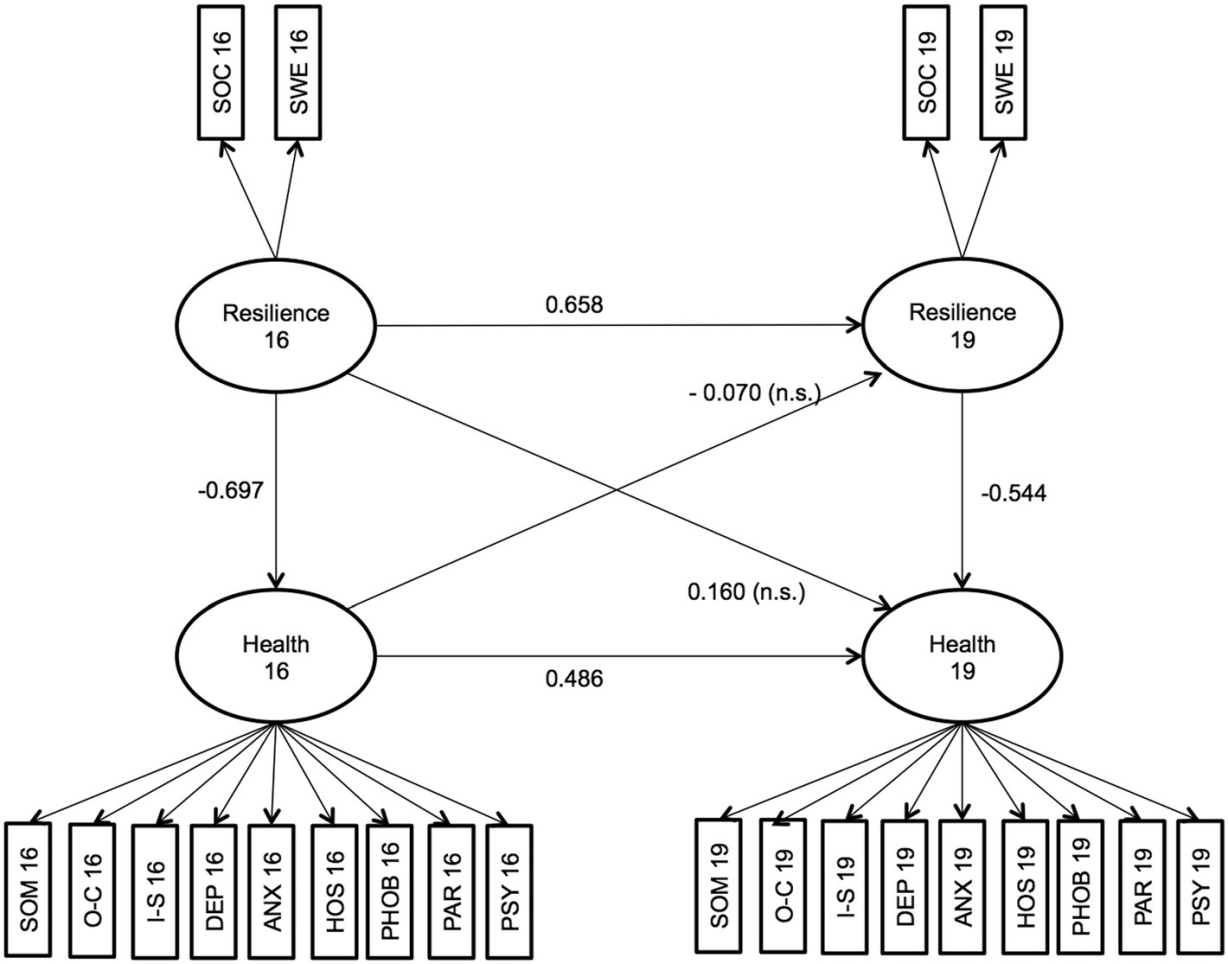
SEM model with all paths.

The first model included all possible paths to fully model the influence across time with resilience at age 19 being regressed on resilience at age 16 and mental health at age 16. Mental health at age 19 was regressed on mental health at age 16 as well as resilience at age 16 and 19. Mental health at age 16 was regressed on resilience at age 16. The full model can be seen in Figure 1. This model fitted the data reasonably well, χ^2^ (204) = 399.56, p = .000, RMSEA = 0.079, CFI = 0.922. The main finding is that resilience significantly influences mental health cross-sectionally (−0.697 at age 16 and −0.544 at age 19). Across time, resilience at age 16 significantly influences resilience at age 19 (0.658). Also mental health at age 16 significantly influences mental health at age 19 (0.486). However, the model does not show any significant mutual influence of the latent variables across time. The influence of resilience at age 16 on mental health at age 19 (0.160) is not significant, the same as the influence of mental health at age 16 on resilience (−0.070) at age 19.

**Figure 2:**
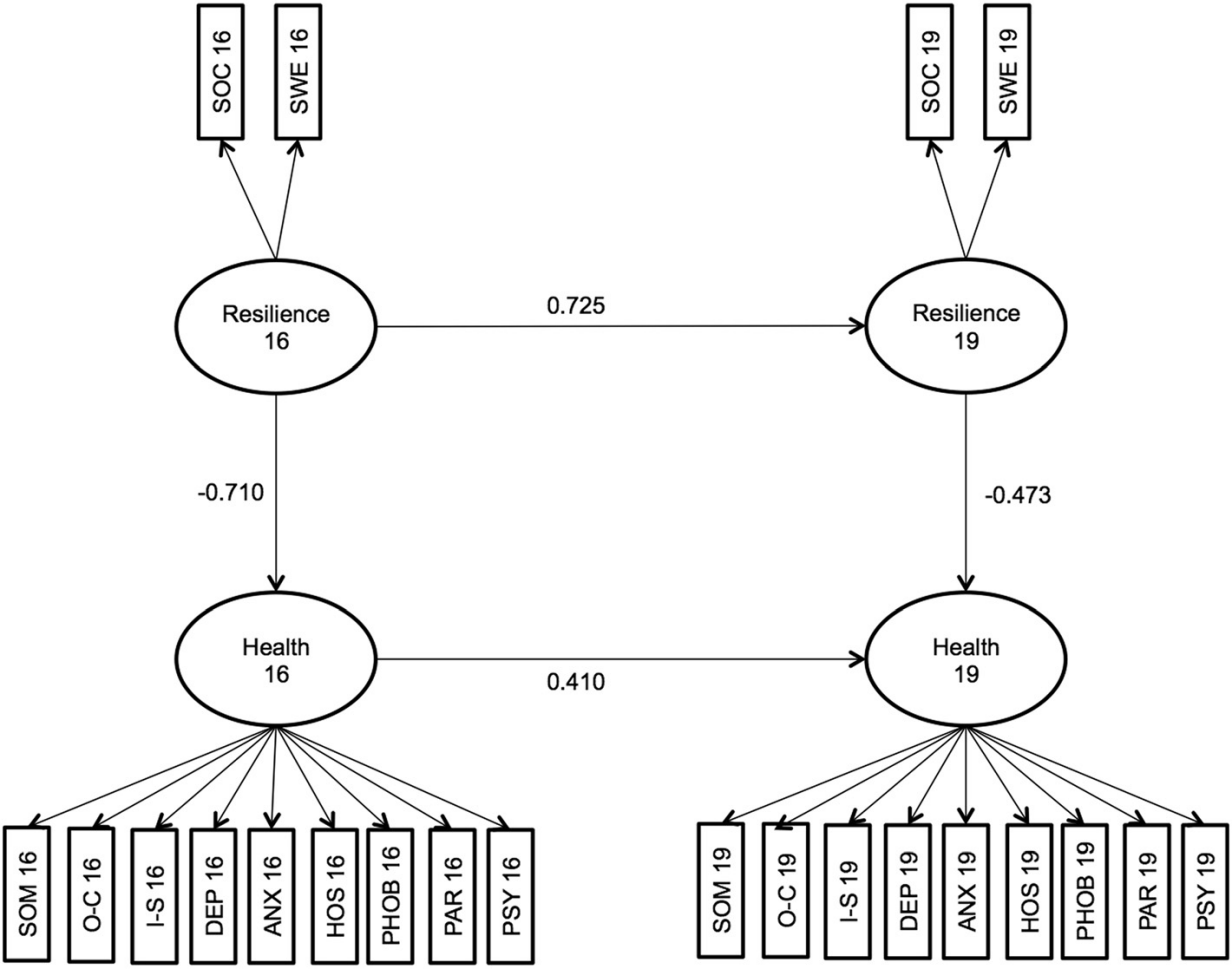
SEM model with only significant paths included.

In the final step, we estimated a second model with only the significant paths of the first model included. Cross-sectionally, health at age 16 was regressed on resilience at age 16 and health at age 19 was regressed on resilience at age 19. Across time, only health at age 19 was regressed on health at age 16 as well as resilience at age 19 on resilience at age 16. The second model can be seen in Figure 2. This model fitted the data slightly better, χ^2^ (206) = 401.977, p = .000, RMSEA = 0.078, CFI = 0.922. Resilience at age 16 significantly influences health at age 16 (−0.710) as well as resilience at age 19 (0.725). Health at age 16 influences health at age 19 (0.410) and resilience at age 19 influences health at age 19 (−0.473).

## Discussion

The aim of this study was to examine similarities of two resilience factors (SOC and self-efficacy) and the mutual influences of these resilience factors and mental health in adolescents.

### SOC and self-efficacy (SWE) are highly related

Our findings provide more evidence for construct validity of sense of coherence and self-efficacy. Both can be modeled as a common resilience factor and positively influence mental health. This provides evidence that SOC (as measured with a 13-item short version of the classical Life orientation Questionnaire) and a generalized self-efficacy (as measured with SWE) are indeed highly related constructs.

### Mental health and resilience factors

Similar to the present study the relationship between mental health and SOC has been confirmed by a number of studies, while its relationship to physical health is – though being present – much less impressing (for an overview see Bengel [36]).

Concerning the mutual influence of resilience and mental health across time, our findings show that resilience is not significantly influenced by prior mental health. Yet people’s self-assessment of mental health and their sense of coherence seem to be closely linked. Additionally our findings support the hypothesis that SOC is already a very stable construct for younger adults. Honkinen et al. [37] have shown that early childhood behavioral problems at age 3 to 12 can predict SOC scores at the age of 18. Similarly it was shown that SOC could be reliably used in a population of 12 year old children [38]. This evidence supports the idea that the foundations of at least some aspects of resilience are rather laid in earlier childhood than in adolescence.

### Age specific development and stability of SOC and self-efficacy

A number of studies have shown that Antonovsky’s expectations about Sense of Coherence being a stable construct in adulthood (at least from the age of 30 onwards) could not be confirmed and it was demonstrated that the SOC often increases with age [39,40]. Yet SOC is also far from fluctuating and begins to show consistent patterns at least in early adolescents. Using mixture modeling with latent classes in a very large sample starting at age 20, Feldt et al. [41] could show that SOC has a general increasing trend, independent of age. They found the greatest stability of SOC in latent classes with a high SOC. Our study results do not contradict those findings. Instead, we can offer insight into the development of SOC in a younger age group.

The invariance of the resilience factors in the present study is astounding, since the age of 16 to 19 is an important time for developing one’s individual identity [42]. Both Sense of Coherence and self-efficacy resemble a person’s view of her own abilities and her view of the world which could be seen as an important aspect of identity.

However, our findings support the idea that SOC and self-efficacy are indeed protective factors which are established in early childhood, rather than in later years.

### The discussion of measurement of sense of coherence

High correlations between SOC and measurements of depression [43,44] and neuroticism [45-47] have led some authors to believe that measuring SOC is dispensable. As neuroticism is considered to be a basic personality trait [48], our findings support the idea that SOC is indeed much more like a personality trait than an orientation in life as claimed originally. SOC certainly shows considerable overlap with negative emotionality or reversed neuroticism and comprises aspects of emotional stability.

While we argue that SOC has explanatory value on its own, we agree to the demand for a more prominent role of emotions, social factors and people’s self-directed activities in the measurement of people’s Sense of Coherence and behaviors in general [49]. We also miss sensitivity for stage specific developmental tasks in Antonovsky’s original Orientation of Life questionnaire.

Likewise self-efficacy appears to be a very stable construct as well. In many cases self-efficacy is adapted to specific contexts, e.g. drinking alcohol [50], but a more generalized self-efficacy might be an important aspect of resilience.

Taken as a whole, the present study provides evidence that SOC and self-efficacy are indeed aspects of resilience that are already quiet stable in adolescence, rather than a partial dimension of current health status.

If the idea of a significant overlap of neuroticism and SOC is taken seriously, improving people’s sense of coherence might be as difficult as changing aspects of neuroticism. Comparable with the debate on personal versus social or cultural resilience we should also expand the conceptualization of salutogenesis from individuals to social systems and their norms or cultural ideas.

If an attempt to influence those global factors is made, it seems reasonable to start early, maybe even as early as in kindergarten [51,52] – and not to focus on individuals only but address group norms and settings.

### Limitations of the study

One aspect that clearly limits the results of the present study is the rather small sample size. For structural equation modeling, a larger sample might lead to better statistical results. This might be an explanation for the rather low model fit in our study as well as possibly our inability to reproduce the three factor structure of the sense of coherence scale.

As the SCL90-R was only provided twice in the whole RISA study, we could only use data from two points in time. It would have been interesting to measure the further development of mental health in early adulthood. Our study however, does include an important developmental phase. About two thirds of the participants in this study had completed school in this phase (at age 19), so we can be sure that participants experienced important changes in their lives.

The measurement of the sense of coherence is also subject to criticism. We had to use an early German adaption of the short 13 item SOC scale, because this particular version was chosen at the planning stage of the 10 year longitudinal study and wasn’t changed later because of consistency of measurement. We therefore have a measurement for which no definite normative scores are available. It might also be problematic that it measures sense of coherence on a 5 point scale, rather than on a 7 point scale. We do not consider this a severe problem as we can show that our scale – adjusted to a 7 point scale – produces scores that are comparable to a later version of the SOC scale for which normative scores are available.

### Further conclusions for research

The stability of SOC in the developmentally changeful age of 16–19 years calls for the measurement of additional resilience factors that are more closely related to youth specific situations and challenges, such as school performance and family stress. Like others we assume the SOC to be too much focused on cognitive phenomena of self-perception that may easily be overwritten by more emotional-situational factors.

## Consent

Written informed consent was obtained from the study participants for publication of this report and any accompanying images.

## Competing interests

The authors declare that they have no competing interests.

## Authors’ contributions

HJ conventionalized and designed the study, organized data acquisition over 10 years. DG has analyzed the sub-dataset statistically. Both have drafted, written and given final approval of the paper.

## Authors’ information

Henrik Jungaberle is a health, prevention and education researcher at the Institute of Medical Psychology, University Hospital Heidelberg. He has developed a risk education program, REBOUND, which builds on resilience education theories.

Dennis Grevenstein is a psychologist at Heidelberg University working on clinical, cognitive and social aspects of human behavior.

## References

[B1] Antonovsky A Unraveling the mystery of health: How people manage stress and stay well 1987 San Francisco, CA US: Jossey-Bass

[B2] Antonovsky A Cooper CL, Payne R The structural sources of salutogenic strengths Personality and stress: Individual differences in the stress process 1991 Oxford England: John Wiley & Sons 67 104 Wiley series on studies in occupational stress

[B3] Antonovsky A McCubbin HI Thompson EA Thompson AI Fromer JE The sense of coherence: An historical and future perspective Stress, coping, and health in families: Sense of coherence and resiliency 1998 Thousand Oaks, CA US: Sage Publications, Inc 3 20 Resiliency in families series, Vol. 1

[B4] Eriksson M Lindström B Antonovsky’s Sense of coherence scale and the relation with health: a systematic review J Epidemiol Community Health 2006 60 376 381 10.1136/jech.2005.041616 16614325 PMC2563977

[B5] Nilsson KW Leppert J Simonsson B Starrin B Sense of coherence and psychological well-being: improvement with age J Epidemiol Community Health 2010 64 347 352 10.1136/jech.2008.081174 19692734

[B6] Gilbar O Relationship between burnout and sense of coherence in health social workers Soc Work Health Care 1998 26 39 49 10.1300/J010v26n03_03 9456473

[B7] Klepp OM Mastekaasa A Sørensen T Sandanger I Kleiner R Structure analysis of Antonovsky’s sense of coherence from an epidemiological mental health survey with a brief nine-item sense of coherence scale Int J Methods Psychiatr Res 2007 16 11 22 10.1002/mpr.197 17425244 PMC6878461

[B8] Lustig DC Strauser DR The impact of sense of coherence on career thoughts for individuals with disabilities Rehabilitation Counseling Bulletin 2008 51 139 147 10.1177/0034355207311313

[B9] von Bothmer MIK Fridlund B ORIGINAL ARTICLE Self-rated health among university students in relation to sense of coherence and other personality traits Scand J Caring Sci 2003 17 347 10.1046/j.0283-9318.2003.00234.x 14629637

[B10] Wiesmann U Hannich H-J Salutogenic perspectives on health maintenance. The role of resistance resources and meaningfulness GeroPsych 2011 24 127 135

[B11] Carstens JA Spangenberg JJ Major depression: A breakdown in sense of coherence? Psychol Rep 1997 80 1211 1220 10.2466/pr0.1997.80.3c.1211 9246887

[B12] Tagay S Mewes R Brähler E Senf W Sense of coherence bei Bulimie-Patientinnen: ein protektiver Faktor für psychische Gesundheit? Psychiatr Prax 2009 36 30 34 10.1055/s-2008-1067461 18645929

[B13] Zirke N Schmid G Mazurek B Klapp BF Rauchfuss M Antonovsky’s Sense of Coherence in psychosomatic patients: A contribution to construct validation GMS Psycho-Social-Medicine 2007 4 1 9 PMC2736525 19742290

[B14] Lengning A Mackowiak K Steinhoff S Franke A Zusammenhänge zwischen Ängstlichkeit, Angstbewältigung und Salutogenese in der Kindheit Zeitschrift für Gesundheitspsychologie 2009 17 151 157 10.1026/0943-8149.17.4.15119349661

[B15] Moksnes UK Espnes GA Lillefjell M Sense of coherence and emotional health in adolescents J Adolesc 2012 35 433 441 10.1016/j.adolescence.2011.07.013 21831417

[B16] Ajzen I The theory of planned behavior Organ Behav Hum Decis Process 1991 50 179 211 10.1016/0749-5978(91)90020-T

[B17] Bandura A Self-efficacy: Toward a unifying theory of behavioral change Psychol Rev 1977 84 191 215 847061 10.1037//0033-295x.84.2.191

[B18] Schwarzer R Self-efficacy: Thought control of action 1992 Washington, DC US: Hemisphere Publishing Corp

[B19] Bandura A Self-efficacy: the exercise of control. 1. print. edn 1997 New York: Freeman

[B20] Kadden RM Litt MD The role of self-efficacy in the treatment of substance use disorders Addict Behav 2011 36 1120 1126 10.1016/j.addbeh.2011.07.032 21849232 PMC3179802

[B21] Tate SR Wu J McQuaid JR Cummins K Shriver C Krenek M Brown SA Comorbidity of substance dependence and depression: Role of life stress and self-efficacy in sustaining abstinence Psychol Addict Behav 2008 22 47 57 18298230 10.1037/0893-164X.22.1.47

[B22] Walker DD Neighbors C Rodriguez LM Stephens RS Roffman RA Social norms and self-efficacy among heavy using adolescent marijuana smokers Psychol Addict Behav 2011 25 727 732 21842969 10.1037/a0024958 PMC3342009

[B23] Shields C Spink K Chad K Odnokon P The confidence to get going: The moderating effects of depressive symptoms on the self-efficacy-activity relationship among youth and adolescents Psychol Health 2010 25 43 53 10.1080/08870440802439065 20391206

[B24] Heitzmann CA Merluzzi TV Jean-Pierre P Roscoe JA Kirsh KL Passik SD Assessing self-efficacy for coping with cancer: Development and psychometric analysis of the brief version of the Cancer Behavior Inventory(CBI-B) Psychooncology 2011 20 302 312 10.1002/pon.1735 20878830

[B25] Egger JW Selbstwirksamkeitserwartung - ein bedeutsames kognitives Konstrukt für gesundheitliches Verhalten Psychologische Medizin 2011 22 43 58

[B26] Abel T Kohlmann T Noack H Noack H Eine deutsche Übersetzung des SOC Book Eine deutsche Übersetzung des SOC 1995 City: Institut für Sozial- und Präventivmedizin

[B27] Schumacher J Gunzelmann T Brähler E Deutsche Normierung der Sense of Coherence Scale von Antonovsky Diagnostica 2000 46 208 213 10.1026//0012-1924.46.4.208

[B28] Schumacher J Wilz G Gunzelmann T Brähler E Die sense of coherence scale von Antonovsky — teststatistische Überprüfung in einer repräsentativen Belvölkerungsstichprobe und Konstruktion einer Kurzskala Psychother Psychosom Med Psychol 2000 50 472 482 10.1055/s-2000-9207 11199111

[B29] Zimprich D Allemand M Hornung R Measurement invariance of the abridged sense of coherence scale in adolescents Eur J Psychol Assess 2006 22 280 287 10.1027/1015-5759.22.4.280

[B30] Hinz A Schumacher J Albani C Schmid G Brähler E Bevölkerungsrepräsentative Normierung der Skala zur Allgemeinen Selbstwirksamkeitserwartung Diagnostica 2006 52 26 32 10.1026/0012-1924.52.1.26

[B31] Schwarzer R Jerusalem M Weinman SWMJE J Generalized Self-Efficacy scale Measures in health psychology: A user’s portfolio Causal and control beliefs 1995 Windsor, UK: NFER-Nelson 35 37

[B32] Derogatis LR Fitzpatrick M Maruish ME The SCL-90-R, the Brief Symptom Inventory (BSI), and the BSI-18 The use of psychological testing for treatment planning and outcomes assessment: Volume 3: Instruments for adults (3rd ed) 2004 Mahwah, NJ US: Lawrence Erlbaum Associates Publishers 1 41

[B33] Franke A BSI - Brief Symptom Inventory von L.R. Derogatis (SCL-90-R) - Deutsche Version. Manual 2000 Beltz: Göttingen

[B34] Franke A SCL-90-R - Die Symptom-Checkliste von Derogatis - Deutsche Version. Manual (2. revidierte und erweiterte Auflage) 2002 Beltz: Göttingen

[B35] Muthén LK Muthén BO Mplus User’s Guide 1998–2007 Fifth Los Angeles, CA: Muthén & Muthén

[B36] Bengel J Was erhält Menschen gesund? Antonovsky’s Modell der Salutogenese - Diskussionsstand und Stellenwert. Eine Expertise 2002 BZgA: Köln: Bundeszentrale für gesundheitliche Aufklärung (Expertise of the German Federal Center for Health Education)

[B37] Honkinen P-L Aromaa M Suominen S Rautava P Sourander A Helenius H Sillanpää M Early childhood psychological problems predict a poor sense of coherence in adolescents: A 15-year follow-up study J Health Psychol 2009 14 587 600 10.1177/1359105309103578 19383659

[B38] Honkinen P-LK Suominen SB Välimaa RS Helenius HY Rautava PT Factors associated with perceived health among 12-year-old school children. Relevance of physical exercise and sense of coherence Scand J Public Health 2005 33 35 41 10.1080/14034940410028307 15764239

[B39] Frenz A Carey M Jorgensen R Psychometric evaluation of Antonovsky’s Sense of Coherence Scale Psychol Assess 1993 5 145 153

[B40] Larsson G Kallenberg K Sense of Coherence, socioeconomic conditions and health Eur J Public Health 1996 6 175 180 10.1093/eurpub/6.3.175

[B41] Feldt T Leskinen E Koskenvuo M Suominen S Vahtera J Kivimäki M Development of sense of coherence in adulthood: A person-centered approach. The population-based HeSSup cohort study Quality of Life Research: An International Journal of Quality of Life Aspects of Treatment, Care & Rehabilitation 2011 20 69 79 10.1007/s11136-010-9720-7 20686925

[B42] Erikson EH Identity and the life cycle 1980 New York, NY US: W W Norton & Co

[B43] Bowman B Cultural pathway towards Antonovsky’s Sense of Coherence J Clin Psychol 1997 53 139 142 10.1002/(SICI)1097-4679(199702)53:2<139::AID-JCLP7>3.0.CO;2-O 9029344

[B44] Kravertz S Drory Y Florian V Hardiness and sense of coherence and their relation to negative affect Eur J Personal 1993 7 233 244 10.1002/per.2410070404

[B45] Feldt T Metsäpelto R-L Kinnunen U Pulkkinen L Sense of coherence and five-factor approach to personality: Conceptual relationships Eur Psychol 2007 12 165 172 10.1027/1016-9040.12.3.165

[B46] Frommberger U Stieglitz R-D Straub S Nyberg E Schlickewei W Kuner E Berger M The concept of ‘sense of coherence’ and the development of posttraumatic stress disorder in traffic accident victims J Psychosom Res 1999 46 343 348 10.1016/S0022-3999(98)00117-2 10340233

[B47] Gibson LM Cook MJ Neuroticism and sense of coherence Psychol Rep 1996 79 343 349 10.2466/pr0.1996.79.1.343 8873826

[B48] McCrae RR Costa PT Jr Pervin LA, John OP A Five-Factor theory of personality Handbook of personality: Theory and research (2nd ed) 1999 New York, NY US: Guilford Press 139 153

[B49] Hurrelmann K Gesundheitssoziologie (Sociology of Health) 2006 Juventa: Weinheim

[B50] Young RM Hasking PA Oei TPS Loveday W Validation of the Drinking Refusal Self-Efficacy Questionnaire–Revised in an Adolescent Sample (DRSEQ-RA) Addict Behav 2007 32 862 868 10.1016/j.addbeh.2006.07.001 16919885

[B51] Krause C Developing sense of coherence in educational contexts: Making progress in promoting mental health in children Int Rev Psychiatry 2011 23 525 532 10.3109/09540261.2011.637907 22272590

[B52] Wiesmann U Krause C Düerkop S Hannich H-J Subjektive Befindlichkeit im Grundschulalter: Eine erste Validierung des ICH BIN ICH Z Med Psychol 2008 17 39 47

